# Supplementary figures and images for: Betel Nut Arecoline Induces Different Phases of Growth Arrest between Normal and Cancerous Prostate Cells through the Reactive Oxygen Species Pathway
Source: Int J Mol Sci. 2020 Dec 3;21(23):9219. doi: 10.3390/ijms21239219 (PMC7729937; doi:10.3390/ijms21239219)

# (A) RWPE-1

24 h

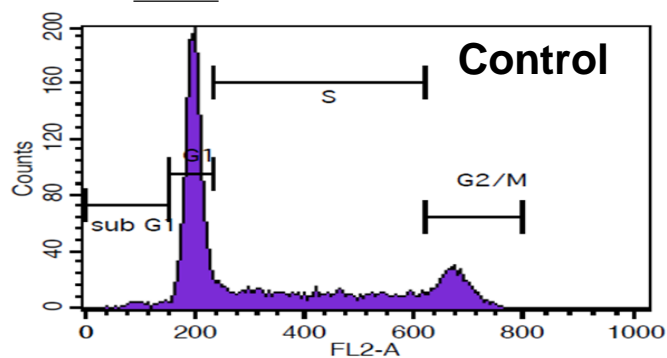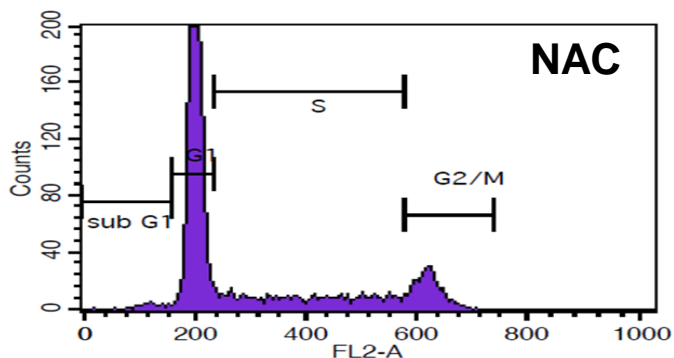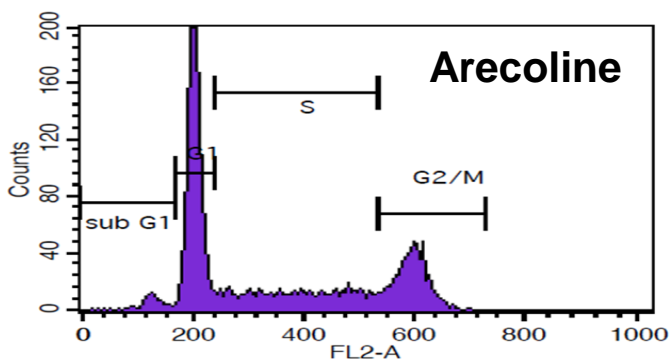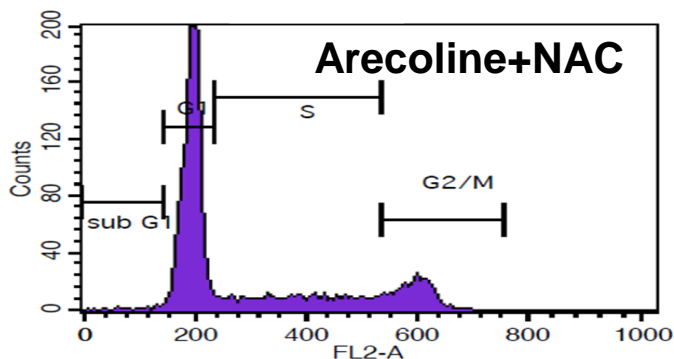

48 h

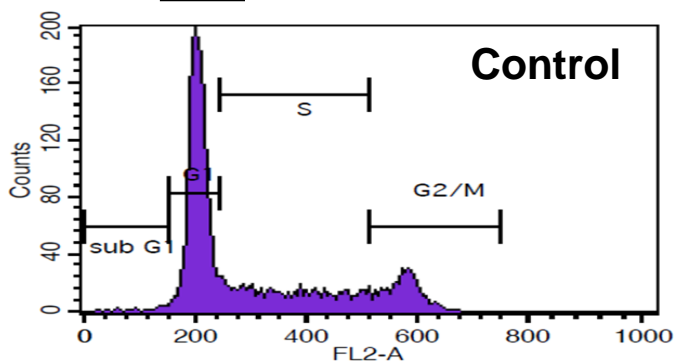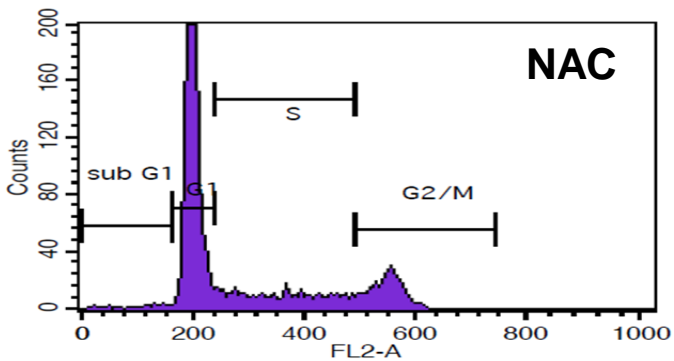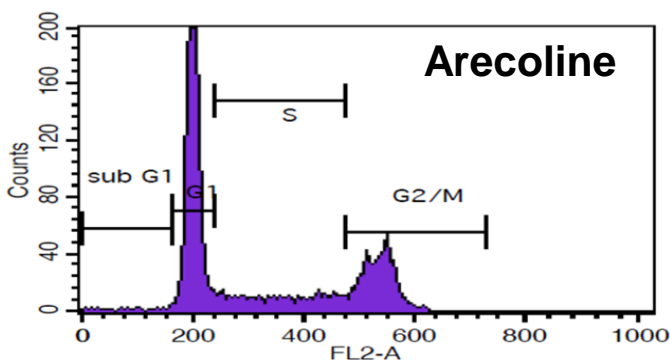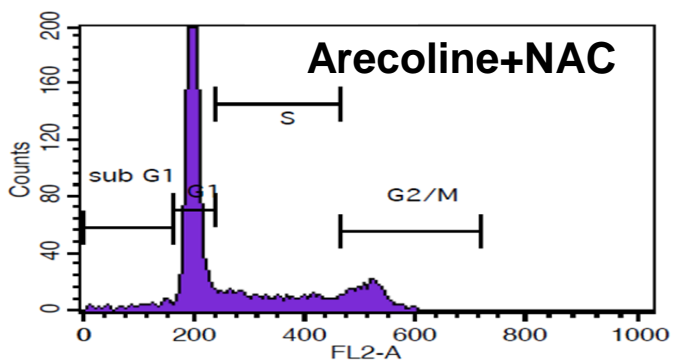

## (B) PC-3

24 h

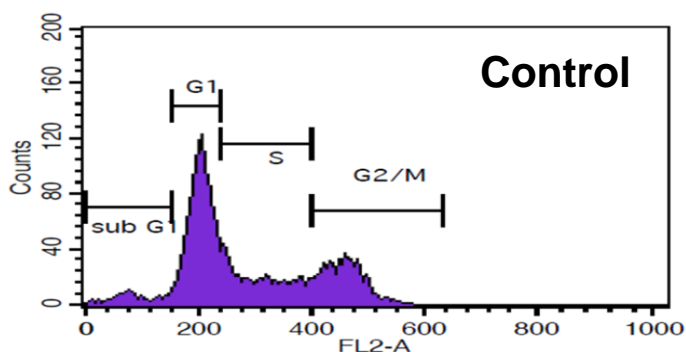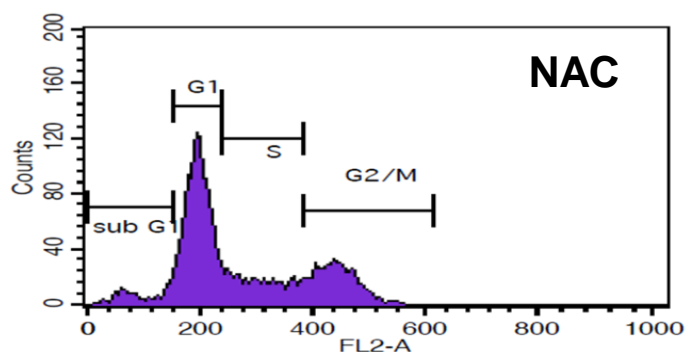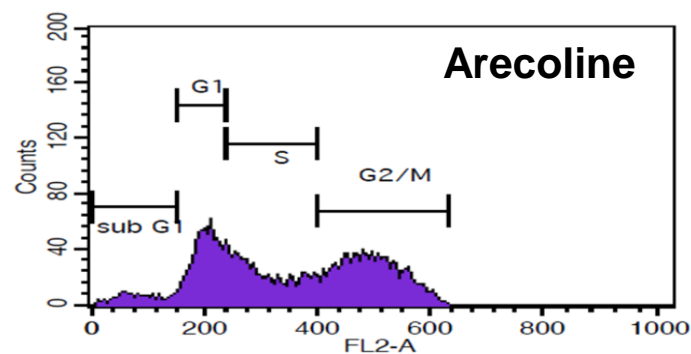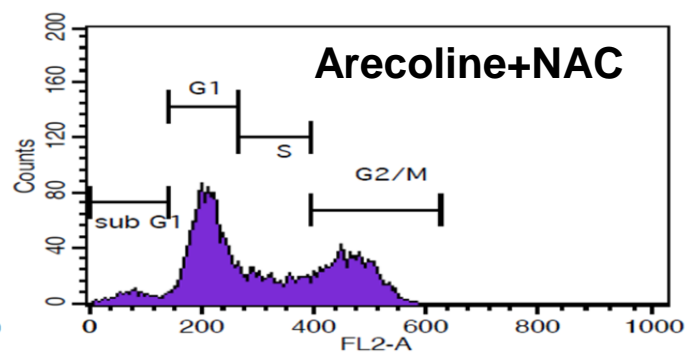

48 h

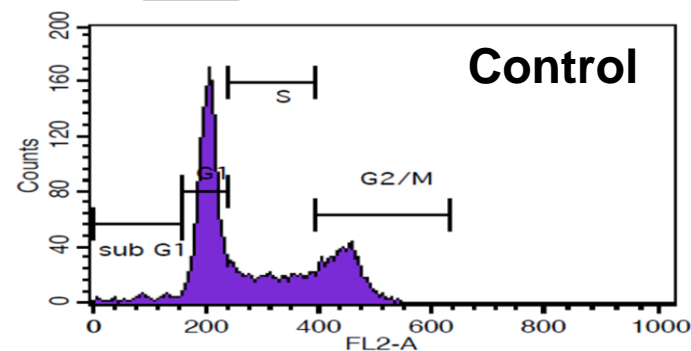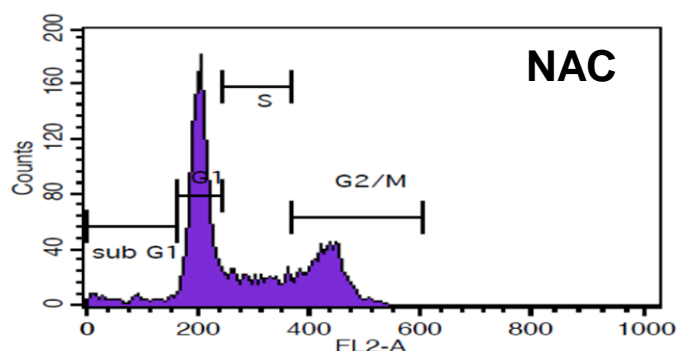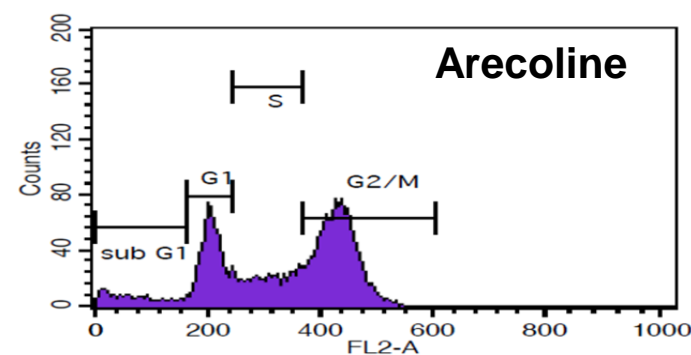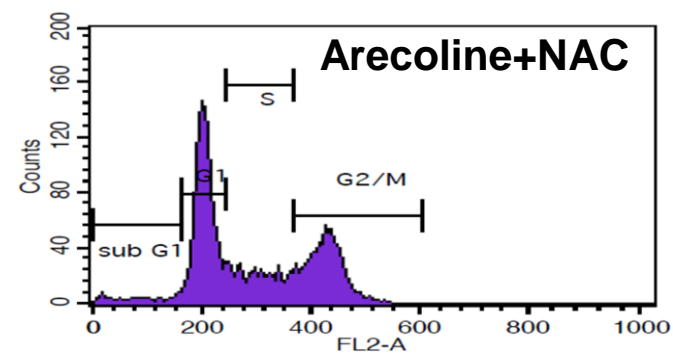

(C) LNCaP

24 h

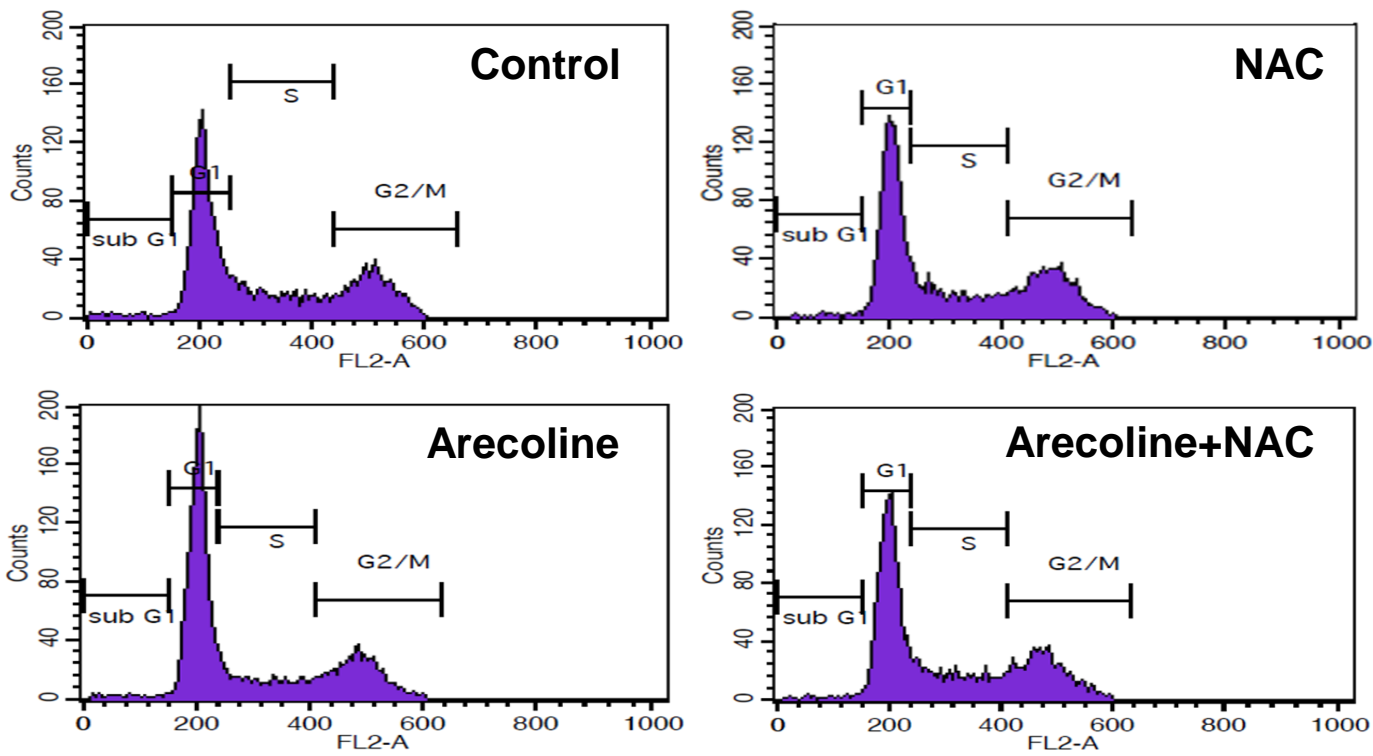

48 h

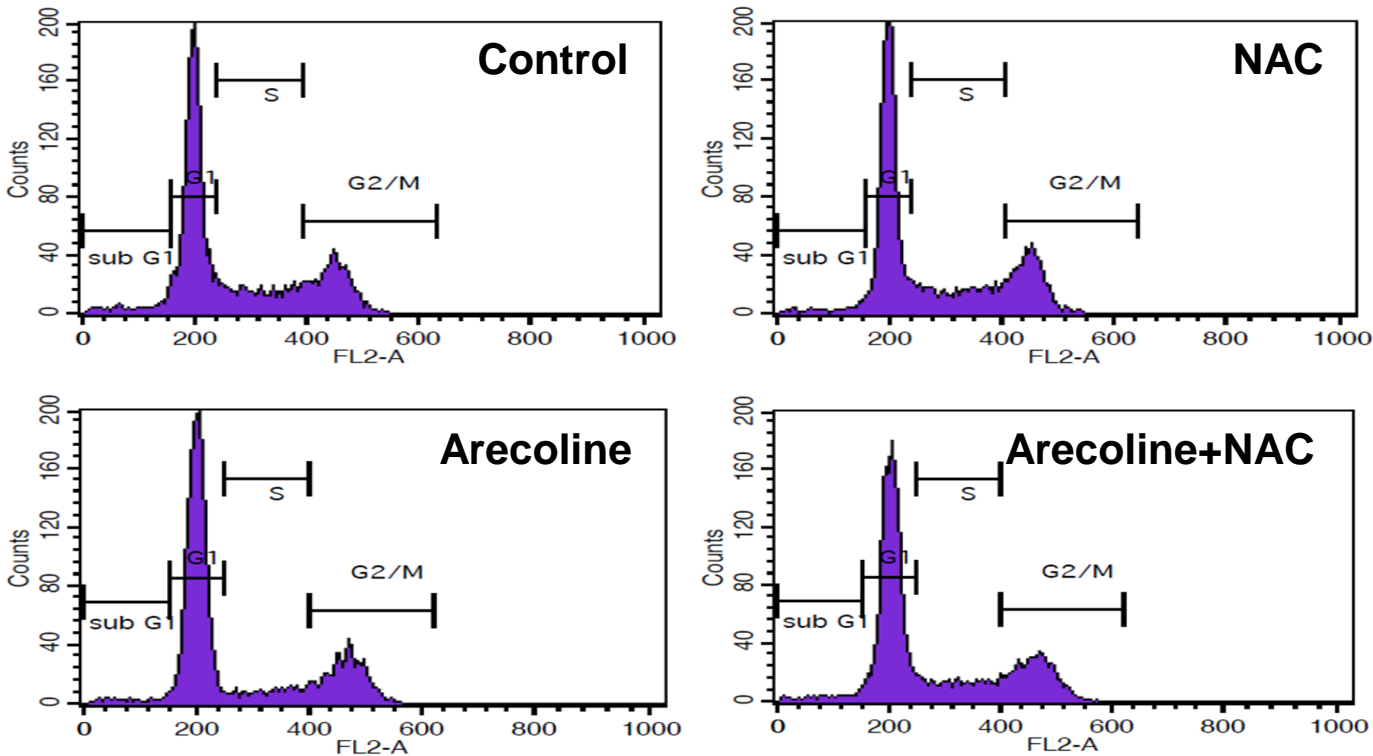

Supplement: Supplementary file 1 [file ijms-21-09219-s001.pdf]
